# Primary health care in the context of the COVID-19 pandemic in 10 South-East Asian countries: a cross-case synthesis with lessons for future health systems strengthening

**DOI:** 10.1136/bmjgh-2024-018076

**Published:** 2025-06-30

**Authors:** Alexandra Edelman, Robert Marten, Ibadat Dhillon, Adithyan Geetha Suresh, Thaksaphon Thamarangsi, John Grundy, Manoj Jhalani, Kumanan Rasanathan

**Affiliations:** 1 Menzies School of Health Research, Charles Darwin University, Alice Springs/ Darwin, Northern Territory, Australia; 2 College of Medicine and Dentistry, James Cook University, Townsville, Queensland, Australia; 3 Alliance for Health Policy and Systems Research, World Health Organization, Geneva, GE, Switzerland; 4 World Health Organization Regional Office for South-East Asia, New Delhi, India

**Keywords:** Health systems, Health policies and all other topics

## Abstract

**Introduction:**

Strengthening primary health care (PHC) in the WHO South-East Asia Region is key to addressing evolving health needs, including the high burden of non-communicable diseases and emerging public health threats within rapidly changing demographic, climatic and geopolitical contexts. Between 2021 and 2023, 10 case studies were conducted to examine PHC in the context of the COVID-19 pandemic. A cross-case regional synthesis aimed to identify key lessons for PHC strengthening from the pandemic experience.

**Methods:**

The synthesis involved comparative analysis using an analytic framework comprising three PHC components framed by the Astana PHC vision: integrated primary care and essential public health functions; multisectoral policy and action; and community empowerment. The case studies used document review and consultations with national PHC experts and policymakers.

**Results:**

*Integrated primary care:* The pandemic crisis prompted health workforce mapping to meet demand, well-coordinated task sharing and shifting between facilities and organisations, and new technology-enabled platforms and models of care to improve healthcare access and continuity. *Multisectoral collaboration:* Multisectoral PHC reforms included expanding the role of multiple sectors to implement public health measures, including testing, contact tracing, border controls and quarantine. New or expanded multiagency and multilevel collaborations involved different government departments coordinating responses across health and other sectors. *Community empowerment:* Active and engaged communities, and community trust in government services and the health system, contributed to positive responses to government-issued messaging and effective mobilisation of community resources. Community engagement platforms created space for community participation in health care decision-making.

**Conclusion:**

Findings demonstrate how PHC principles remain relevant not only for responding to public health emergencies, but also for improving and promoting health system resilience. Findings highlight opportunities to further examine and implement health workforce, community engagement, digital technology and governance strategies to meet evolving epidemiological and climate-related health challenges facing the Region.

**WHAT IS ALREADY KNOWN ON THIS TOPIC**

- Regional high-level commitments to primary health care (PHC) highlight its continued importance for achieving universal health coverage, the health-related Sustainable Development Goals, as well as ensuring health security and health system resilience.
- The WHO South-East Asia Region, in the context of scarce resources, has a long history of PHC-related innovations.
- COVID-19 pandemic responses reveal lessons to accelerate progress to expand PHC.

**WHAT THIS STUDY ADDS**

**HOW THIS STUDY MIGHT AFFECT RESEARCH, PRACTICE OR POLICY**

- Countries’ experiences offer insights into health workforce, community engagement, digital technology, governance and financing strategies that can inform efforts to strengthen the Region’s health systems.
- Despite continued high-level PHC commitments, policymakers require additional resources and support for implementation. Institutionalising learning health systems could help accelerate efforts and will require strengthened subnational, national and international knowledge management and translation platforms

## Introduction

 Strengthening primary health care (PHC) in the WHO South-East Asia Region is key to addressing evolving health needs, including the high burden of non-communicable diseases (NCDs) and emerging public health threats within rapidly changing epidemiologic, demographic, climatic and geopolitical contexts.^1^ Countries in the Region—Bangladesh, Bhutan, Democratic People’s Republic of Korea (DPR Korea), India, Indonesia, Maldives, Myanmar, Nepal, Sri Lanka, Thailand and Timor-Leste—represent more than a quarter of the world’s population and are key drivers of global economic growth. The COVID-19 pandemic challenged health systems, drawing increased attention to the role of PHC in a pandemic context as well as in responding to the Region’s growing burden of NCDs, persistent health inequities, high rates of poverty, ageing populations, rapid urbanisation and vulnerability to health emergencies.^1^ Climate and environmental change will exacerbate the health challenges, with the Region being among the most vulnerable to increasingly intense and unpredictable weather events, heat waves, droughts and rises in sea levels.^2^

The Region’s diversity provides an opportunity for assessing and contrasting the applicability of PHC approaches in a wide set of contexts. The 11 countries are diverse in geography, culture, population size and political organisation, with some countries undergoing rapid economic growth, while others have been exposed to protracted humanitarian emergencies (Myanmar, DPR Korea). The universal health coverage (UHC) Service Coverage Index (SCI), derived from indicators in four domains (reproductive, newborn, maternal and child health; infectious diseases; NCDs; and service capacity and access), provides a measure of a country’s ability to provide essential health services.^3^ The average SCI coverage rate for the Region has increased from 47 to 62 between 2010 and 2021, though estimates for 2021 range from 46 in Timor-Leste to 82 in Thailand.^3^ Progress remains slow: the Region’s UHC SCI target of 80 is unlikely to be met by 2030 with current rates of progress.^3^ As most UHC interventions reflect a PHC approach to deliver essential services and reduce health inequities, continued efforts to advance PHC-focussed policies and actions are critical.

While the Region has a long history of both national and regional commitments to PHC (see tables1  2), specific reform priorities and implementation vary across countries.^4^ The 2023 Delhi Declaration on Strengthening PHC,^5^ recognising the opportunity and imperative to transform health systems against the backdrop of COVID-19, expresses health ministers’ reaffirmed commitments to PHC (table 1). The Declaration gave further impetus to the Region’s commitment to establish learning health systems at national and subnational levels, supported by the 2022 South-East Asia Regional Forum for PHC-Oriented Health Systems (SEAR PHC Forum).^6^

**Table 1:** Political commitments to PHC within the WHO South-East Asia Region since COVID-19

Declaration by the Health Ministers of Member States at the Seventy-fourth Session of the WHO Regional Committee for South-East Asia, 2021(SEA/RC74/R1)	Addresses the challenge of COVID-19 and articulates measures to ‘build back better’ essential health services to achieve UHC and the health-related SDGs. Recognises a ‘once-in-a-century’ opportunity to advance transformation towards resilient PHC-oriented health systems as the means to achieve population health, well-being and prosperity in the region. Highlights the need for whole-of-government, whole-of-society and health-in-all-policies approaches.
WHO South-East Asia Regional Committee Resolution on Enhancing social participation in support of Primary Health Care and Universal Health Coverage, 2022(SEA/RC75/R3)	Endorses the South-East Asia Regional Strategy for Primary Health Care: 2022–2030 (SEAR PHC Strategy); the SEAR PHC Strategy outlines seven values (universality; equity; solidarity; accountability; people-centredness; resilience and adaptiveness; and evidence-driven action) and 12 strategic actions that aim to advance the Astana PHC components. Action 12 seeks to encourage efforts to institutionalise learning health systems. Requests the establishment of the South-East Asia Regional Forum for PHC-oriented Health Systems (SEAR PHC Forum), bringing together Member States and key PHC-focused development, implementation and academic partners in the region to collectively capture operational learning and strengthen synergy in action.
Relevant major heads of state and government commitments, 2023: United Nations General Assembly Resolution: political declaration of the high-level meeting on UHC, October 2023 (global; co-chaired by Thailand) G20 New Delhi Leaders’ Declaration, September 2023 (led by India)	Recognises the fundamental role of PHC in achieving UHC and the health-related SDGs, as well as the Declaration of Astana. Commits countries to strengthen PHC, including to strengthen PHC, health workforce and health systems to better than pre-pandemic levels within 2–3 years.
Delhi Declaration on Strengthening PHC, 2023(SEA/RC76/R3)	Reinforces strong direction from heads of state and government and recognises responsibility to strengthen PHC as the cornerstone to advance the UHC agenda and the health-related SDGs through a set of 12 commitments, including strengthening subnational, national and cross-country systems for knowledge management and collaboration.

Source: Adapted from refs.^542^,^45^

PHC, primary healthcare; SDGs, Sustainable Development Goals; UHC, universal health coverage.

**Table 2:** Select PHC policy commitments in WHO South-East Asia Region countries

Bangladesh	National Health Policy 2011; Fifth Health, Population, and Nutrition Sector Programme (HPNSP) 2024–2029 with focus on PHC
Bhutan	National Health Policy 2011 (under revision); Service with Care and Compassion Initiative, 2018 (integration of NCDs into PHC); Thirteenth Five Year Plan, 2024–2029 with focus on PHC
DPR Korea	Primary Health Care Strategy 2021–2025
India	National Health Policy 2017 with focus on PHC; Ayushman Bharal—Comprehensive PHC program through health and wellness centres, 2018; Ayushman Barat—Digital Health Mission 2021; Ayushman Barat Health Infrastructure Mission, 2021
Indonesia	Health System Transformation 2022; *Transformasi Layanan Primer* (Primary Service Transformation) as the first pillar of the country’s health transformation; National PHC Integration and the Health Omnibus Law 2023 together driving PHC-oriented reforms
Maldives	Maldives National Health Master Plan 2016–2025; Maldives launched the PHC Demonstration Site in Faafu Atoll in 2022, with the aim to reorient health systems to comprehensive PHC and with ongoing expansion to 10 atolls
Myanmar	National Health Plan 2017 (under revision)
Nepal	Constitution of Nepal 2015 (guaranteeing right to free basic and emergency health services); Public Health Service Act 2018 and National Health Policy 2019 to assure constitutional guarantee
Sri Lanka	Policy on Health Care Delivery for UHC 2018; PHC Reforms in progress based on shared-care cluster approach
Thailand	Thailand PHC Act 2019; Public health transformation 2024, from ‘Treats all Diseases’ to ‘Treatment Anywhere’
Timor-Leste	National Health Sector Strategic Plan 2011–2030, 2024 Integrated Health Services Policy with focus on comprehensive PHC

Source: Adapted from refs.^5 46^

NCD, non-communicable disease; PHC, primary healthcare; UHC, universal health coverage.

To support these efforts by identifying lessons for PHC reform from the COVID-19 experience, the Alliance for Health Policy and Systems Research (the Alliance), a WHO-hosted partnership, commissioned nearly 50 country case studies, including 10 conducted between 2021 and 2023 in the South-East Asia Region: Bangladesh, Bhutan, DPR Korea, Indonesia, Maldives, Myanmar, Nepal, Sri Lanka, Thailand and Timor-Leste (note: a case study from India was not commissioned as other studies were already underway).^7^,^16^ The published case studies examine PHC against the three PHC components framed by the 2018 Astana PHC Vision: (1) integrated primary care and essential public health functions; (2) multisectoral policy and action; and (3) community empowerment.^17^ This article presents a synthesis of cross-case findings from the Region’s case studies to address the question: what key lessons from the COVID-19 experience can inform PHC strengthening to build more resilient health systems and accelerate progress towards UHC?

## Methods

10 in-country research teams were contracted to conduct a case study examining PHC in the context of the COVID-19 pandemic. Each case study was published on the Alliance website.^7^,^16^ Detailed case study methods are reported in the individual published reports. Each case study used a consistent two-part approach of conducting: (1) desk-based review of PHC and COVID-19-related academic literature, grey literature such as policy documents and guidelines, and media reports; and (2) stakeholder consultations with PHC experts and high-level policymakers, identified through purposive and snowball sampling. The in-country research teams analysed data thematically using the three PHC components framed by the Astana vision (integrated primary care and essential public health functions, multisectoral policy and action, and community empowerment)^17^ which are reflected in the case study report structures. The case studies also report lessons learnt to improve PHC in the pandemic context, including to strengthen future pandemic preparedness.

To conduct a multicountry case study synthesis, we used a cross-case comparative analysis approach.^18^ This commenced with describing key features of each country context and comparing characteristics and approaches against an analytic framework with broad headings.^18^ As in the individual case studies, the analytic framework was based on the three Astana PHC components modified to reflect the COVID-19 context. Narrative and numerical data extraction from the case studies, conducted by one researcher (AE), used a spreadsheet template (online supplemental file 1) with the following extraction fields: country context (including demographic and epidemiological characteristics), Astana PHC components (three fields with subheadings) and analytic categories to capture reported innovations, enabling factors, barriers and gaps, and recommendations. We added extraction fields corresponding to the PHC strategic and operational levers from the WHO PHC Operational Framework (political commitment and leadership; governance and policy frameworks; funding and allocation of resources; models of care; primary care workforce; physical infrastructure; medicines and other health products; engagement with private sector providers; engagement of community and stakeholders from all sectors; purchasing and payment systems; digital technologies for health and PHC-oriented research)^19^ to draw out more detail on key aspects of PHC. Data analysis used a deductive thematic analysis approach^20^ using the modified Astana PHC headings as themes (table 3). Within each theme, three researchers (AE, RM and JG) used inductive coding to identify seven subthemes, which involved identifying and grouping key concepts in the extracted data and mapping them against the broader analytic framework.

**Table 3:** Analytic framework used to compare and report cross-case findings in the synthesis and inductive themes

Astana primary health care component (modified for COVID-19 context)	Inductive subthemes
Integrated primary care and essential public health functions in the COVID-19 context	Models of care Health workforce Referrals and medication Digital technologies for health
Multisectoral policy and action in the COVID-19 context	Governance and policy frameworks
Engaging and communicating with communities effectively and leveraging community resources in the COVID-19 context	Government-led communication and coordination of public information Engagement of community and other stakeholders

## Results

This section summarises the results against the three modified Astana PHC components in the COVID-19 context and inductive subthemes, commencing with (1) integrated primary care and essential public health functions; followed by (2) multisectoral policy and action; and 3) community engagement.

### Integrated primary care and essential public health functions in the COVID-19 context

#### Models of care

Integrated models of care refer to those that promote continuous, comprehensive and coordinated care, rather than focusing on specific diseases.^19^ Models of primary care vary across the Region’s countries and are delivered through government-funded health care facilities at the local level (eg, community clinics in Bangladesh^7^ and island-level health centres in the Maldives^11^), a growing private health sector (eg, Myanmar^12^) and other non-government organisations. Case studies describe persisting gaps in integrated care resulting in barriers to health care access, including health workforce shortages and maldistribution, a largely unregulated private sector, low levels of government health expenditure, hospital-based health care models and insufficient investment in prevention or attention to the social determinants of health.

Systems enabling delivery of essential public health functions vary. While promotive, preventive and curative care functions are integrated in Indonesia’s *puskesmas* (government-funded primary care units at the subdistrict level),^10^ hospitals and health centres in the Maldives have a dedicated public health unit that provides preventive and promotive services to the community.^11^ Dedicated Hygiene and Anti-Epidemic Health Stations also operate at central, province and county levels in DPR Korea to manage epidemiology and communicable disease response functions.^9^ In Sri Lanka, a national system of integrated public health programmes involve preventive primary care staff assigned to geographically defined populations, supported by networked surveillance and epidemiology services.^14^ Community health workers (CHWs) play a similarly key role in Myanmar in leading nutrition, education, vaccinations, antenatal and postnatal care, and testing and treatment services for communicable diseases and NCDs.^12^

From the start of the COVID-19 pandemic in early 2020 and through 2021, several countries saw a decrease in primary care service utilisation and coverage and a scaling back of other (non-COVID-19) public health functions. For example, Bangladesh saw a fall in essential maternal health service utilisation,^7^ Sri Lanka experienced reduced patient utilisation of public and private primary curative care services^14^ and Myanmar and Nepal both reported reduced immunisation rates.^12 13^ Disconnected emergency responses from primary care planning may have contributed to diminished integrated primary care and essential public health functions as scarce financial and human resources were redirected to the immediate COVID-19 response. In the Maldives, for instance, referrals to the Greater Male Area were restricted to emergency medical care and COVID-19 containment, with patients experiencing delays to care and disruptions to routine surgeries, diagnostic services and inpatient treatment.^11^

The introduction of movement restrictions and provider and community fear of COVID-19 transmission also contributed to disrupted health care access. In Timor-Leste, despite *TramKom* (a community initiative established to transport community members in need of urgent medical attention given limited ambulatory services), many patients struggled to access health services because of suspended public transportation.^16^ Thailand’s mobility restrictions and COVID-19-related staff shortages contributed to reduced NCD screening.^15^ In Nepal, as in other countries, despite government directives and guidelines on the separation of COVID-19 and non-COVID-19 care, many hospitals halted regular outpatient and some inpatient clinics due to uncertainties and fear of COVID-19 transmission.^13^

The role of primary care in supporting public health emergency responses varied across countries and over the course of the pandemic. At least initially, primary care was bypassed as COVID-19 testing and treatment largely took place in hospitals (including designated COVID-19 hospitals established in multiple countries). Primary care services became more involved as case numbers increased and as hospitals became overwhelmed with cases. For example, Indonesia’s network of *puskesmas* took on a central role in testing, contact tracing and treatment with affiliated hospitals and laboratories.^10^ Sri Lanka’s primary care facilities also became more involved as demand increased for emergency services despite initial attempts to separate COVID-19 patients from facilities providing essential care for non-COVID-19 patients.^14^

Several countries established new regional or local facilities specifically for COVID-19 testing. In Bhutan, 55 ‘flu clinics’ were established across all 20 districts and municipalities to support testing.^8^ These were located away from hospitals providing routine services to reduce transmission risks. Four new regional COVID-19 treatment centres in the country pooled resources and clustered case management.^8^ Myanmar similarly established ‘fever clinics’ in schools and community centres, staffed by private physicians and volunteers under Ministry of Health guidance.^12^ Dedicated COVID-19 treatment centres, managed in the municipalities, also came to play a key role in Timor-Leste for asymptomatic, mild and moderate cases (although, initially, confirmed cases were transferred to dedicated treatment centres in the capital, Dili).^16^ In contrast, household doctors in DPR Korea regularly examined people with fever or respiratory symptoms in their village or town.^9^ While coordination of public health and clinical responses was enabled in some countries by establishing dedicated COVID-19 focal points and formal and informal networks, structural fragmentation of services inhibited coordination between service providers (eg, a disconnect between doctor-led flu clinics and CHW-led public health units in the Maldives^11^). Coordinated responses were also hampered by changing guidelines, lack of transparency in the channelling of funds and limited monitoring of resourcing and impacts. Poor role clarity between organisations and levels of government contributed to gaps in services for vulnerable groups.

#### Health workforce

Like other global experiences, several of the Region’s countries experienced an overstretched workforce against a backdrop of shortages and inadequate skill mixes, exacerbated in some cases by workforce burnout over the course of the pandemic. Thailand experienced shortages of infectious disease, critical care and other specialists, as well as effects on staff morale due to fear of infection and high workloads.^15^ In the Maldives, the pandemic drew attention to existing shortages of mental health and rehabilitative providers, with the mental health workforce in the Greater Male Area becoming overwhelmed as reports of mental health issues and domestic violence increased during movement restriction periods.^11^ To address these workforce and capability challenges, three main health workforce strategies emerged from the case studies: task-shifting, re-deployment of the existing workforce and mobilisation of a community-based workforce.

#### Task-shifting

Countries used strategies such as task sharing and shifting between workforce cadres to support the high volume and breadth of the required response. The Bhutan Ministry of Health mapped health workforce clusters to meet surge demands, recalling doctors undergoing postgraduate training outside the country and recruiting recent medical graduates and final-year nursing students to health services.^8^ The Maldives amended licensing guidelines to enable biotechnologists to conduct PCR tests and train school health workers and medical, nursing and health sciences students in sampling techniques.^11^ Sri Lanka implemented task-shifting across sectors such as the police, the military and public administration to support quarantine activities, community mobilisation and vaccinations.^14^

#### Re-deployment of existing workforce

Health care facilities also moved staff between services. For example, hospitals in Sri Lanka reassigned health workers from low-workload settings to busier wards and administrators authorised the reassignment of health staff from low-transmission to high-transmission areas within districts and provinces.^14^ Health workers in Nepal were similarly deployed between districts and facilities.^13^ To minimise the risk of staff shortages due to quarantine directives, hospitals in Thailand established rotation rosters to separate COVID-19 health care teams from staff in other wards.^15^ The pandemic also revealed critical training gaps and opportunities to improve public health education across workforce cadres, such as an opportunity to introduce a standardised curriculum on public emergency preparedness and PHC in Timor-Leste.^16^

#### Mobilising the community-based workforce

The pandemic highlighted the pivotal roles of workforce groups beyond traditional cadres (ie, doctors, nurses and pharmacists). Among the most important roles enabling pandemic responses were medical and health assistants, paramedic staff and various groups of CHWs and volunteers. CHWs in the Maldives supported teams by taking samples, conducting preliminary check-ups, starting treatment and monitoring patients.^11^ Volunteer roles and contributions ranged from formalised roles in the health workforce to informal caring functions delivered in communities, drawing on significant extant social capital. Formal roles included 1350 Village Health Workers in Bhutan who received government training in case recognition, surveillance, reporting and health advocacy.^8^ Similarly, Village Health Volunteers (VHVs) in Thailand, already experienced in infectious disease surveillance, were instrumental in supporting screening, contact tracing and isolation efforts.^15^ VHVs supported the establishment of surveillance teams as part of the pandemic response in all subdistricts. These teams educated communities about the disease, preventive measures and relevant symptoms to self-monitor and report; and enabled monitoring and support of people undergoing self-quarantine. In the Maldives, volunteers were trained and posted within the health emergency operations centre to provide psychological first aid and basic counselling to those in isolation or quarantine.^11^ The medical community not affiliated with government services also volunteered to work at the health emergency operations centre and contribute to the response.^11^ Volunteers were also mobilised in Myanmar—people with a medical background and CHWs were trained and engaged to triage at fever clinics and support the management and coordination of quarantine centres.^12^ Less formally, retirees and family members were involved: Nepal’s army deployed retired medical staff^13^; and, in Timor-Leste, family members provided care for patients who needed continued medical assistance at home.^16^

#### Referrals and medication

Countries used innovative referral and medication dispensing approaches to enable continuity of chronic disease management. Thailand’s VHVs identified and referred patients with chronic conditions to secondary care providers where they were unable or unwilling to visit primary care health facilities.^15^ In some communities in Myanmar, pharmacies dispensed several months of medication to patients with chronic conditions to reduce the frequency of visits to clinics.^12^ Bhutan’s National Contingency Plan integrated multiple strategies to secure provision of emergency services, mobile clinics and medicine refills, continuation of emergency surgeries and cessation of any aerosol-generating procedures.^8^ Patients accessed health services during periods of movement restriction using transport services for those requiring emergency medical care, via teleconsultations by the national referral hospital and via mobile clinics to deliver medications.^8^

#### Digital technologies for health

Countries scaled up or introduced new digital technologies to support COVID-19 surveillance and response. In Bhutan, digital applications (including the COVID-19 Integrated Influenza Surveillance System, the Health Facility System, the *Druk Trace* app, the Quarantine Management System, the Stay Home app and the Check Post Management System) strengthened the government’s response planning, contact-tracing and surveillance efforts.^8^ Bangladesh monitored epidemiological trends using a new dedicated COVID-19 surveillance system and COVID-19 dashboards.^7^ The Maldives similarly introduced a digital information platform to support emergency response planning; this required continuous monitoring and maintenance but ultimately improved information flows to support evidence-informed decision-making.^11^

The crisis also prompted the development of new technology-enabled strategies and models of care to improve health care access and continuity (eg, teleconsultations). In Indonesia, maternal health and antenatal services were delivered using telemedicine through phone communication or, in regions with limited technology access, face-to-face appointments with enhanced infection prevention and control measures.^10^ Telehealth services were essential to reduce unnecessary visits to primary care clinics while meeting the needs of patients and protecting individuals; during the pandemic, these services were covered by the public insurance provider.^10^ In Sri Lanka, the government established a mechanism for home care services that was linked to a dedicated 24/7 call centre for selected districts with a high prevalence of cases.^14^ In the new service model, a designated doctor was accountable for each patient under the supervision of a specialist family physician.

### Multisectoral policy and action in the COVID-19 context

#### Governance and policy frameworks

As pandemic responses were necessarily multifaceted, countries’ public health responses involved multiple sectors in new or scaled up multiagency and multilevel collaborations across government departments. Governments rapidly established high-level coordination committees, policy directions and guidelines to support action. The head of state, prime minister or health minister generally chaired high-level coordination and oversight committees supported by operational and technical committees comprising experts from different fields to advise government policy. Education, defence, housing and other social services collaborated to implement public health measures, including testing, contact tracing, border controls and risk communication. Collaborations involved public and private health services and non-governmental organisations, civil society organisations (CSOs), religious groups, armed forces and community volunteers. Because of the impacts of pandemic responses on peoples’ livelihoods, policies in multiple countries also reflected a dual imperative to protect health and reduce economic impacts. Indonesia’s policy response throughout 2020 and 2021, for example, included four components: (1) strengthening the health sector; (2) protecting community groups and business; (3) reducing pressure on the financial sector and (4) restoring economic resilience and people’s livelihoods as part of a ‘new normal’.^10^ Some policy frameworks aimed to mitigate impacts on vulnerable groups, like informal workers, that were disproportionately affected by movement restriction policies.

Across the countries, centrally coordinated actions of executive government, coupled with decentralised decision-making structures, transparency in how the use of government funds were communicated publicly and development of government-issued guidelines to coordinate crisis responses were key enablers of effective responses. Indonesia’s ‘cluster approach’ enabled whole-of-government planning, supporting the country’s COVID-19 multisectoral response across health care, community engagement, logistics, food security, socio-economic impacts, essential services and protection of vulnerable groups.^10^ Established in 2014, the approach coordinates responses and resources across health, agriculture, domestic affairs, infrastructure, education, religious affairs, and social affairs ministries and search and rescue agencies.^10^ An example of a decentralised approach, Nepal’s newly federalised governance structure facilitated provincial and local government oversight of basic health service provision through community-level health posts and primary care centres.^13^ The pandemic also catalysed the establishment of new cross-ministry planning and operational structures, such as Nepal’s COVID-19 Crisis Management Centres formed at provincial, district and local levels to coordinate across defence, home affairs, federal affairs, health and industry portfolios.^13^ The crisis also revealed opportunities to strengthen governance and policy frameworks, such as in Bangladesh, where disconnected planning between government, private and non-health actors, and between central and subdistrict regions, highlighted the need to strengthen clarity in regulatory and accountability relationships for integrated emergency responses.^7^ Moreover, the case studies draw attention to the need for mechanisms to sustain and strengthen impactful collaborations after emergency responses are scaled down, such as when COVID-19 ceased to be a political priority.

### Community engagement and empowerment in the COVID-19 context

#### Government-led communication and coordination of public information

Government bodies played a vital coordination role in communicating with the public—such as Sri Lanka’s health promotion bureau in the Ministry of Health, which was designated as the country’s risk communication focal point.^14^ The government’s ‘new normal’ campaign, launched in April 2020, educated the community on public health behaviours, seeking to improve awareness among civil society to reinforce existing knowledge and shape attitudes and practices, especially in public activities and work settings, to limit virus spread.^14^ Often led by health ministries with varying involvement of non-government and community sector stakeholders, governments used television, radio, newspapers and social media (including Facebook, Twitter, Instagram, LINE, YouTube, Viber and TikTok) to share information. Television broadcasts shared information about COVID-19 case numbers and government restrictions and provided an opportunity to share best practices and innovative approaches.^14^ Public–private sector collaboration enabled the development of new mobile phone apps.

Challenges for governments in communicating with the public included declining public trust in government messages over time (eg, the Maldives, Nepal^11 13^). In some population groups, waning trust in government authorities resulted in the discontinuation of community-led messaging and peoples’ concealment of COVID-19 symptoms and cases. Further challenges included poor communication infrastructure and low literacy, particularly in rural areas, coupled with community anxiety generated by daily news clips. Disinformation was also circulated on social media about false and ineffective COVID-19 treatment remedies, undermining government messages and requiring careful government monitoring of online platforms (eg, Nepal, Sri Lanka, Thailand^13^,^15^).

While highly centralised communication approaches were sometimes poorly contextualised to local populations, impacting public understanding of the pandemic and response measures, governments made efforts to target communications. Indonesia established ‘COVID-19 alert villages’ to support targeted community-led education about COVID-19 risks and ways to prevent transmission at the local level.^10^ In the Maldives, messages were delivered on different platforms and in different languages.^12^ Nepal similarly developed behaviour change messages in multiple languages to engage communities to promote public health safety measures, encourage quarantine and isolation to prevent transmission, and reduce COVID-19-related stigma and discrimination.^13^ Health officials in Sri Lanka worked closely with specific groups (urban, rural, religious and by occupation) to address their specific needs, and posters were displayed with a checklist to encourage daily behaviours and practices to limit virus transmission within households.^14^ The government also organised musical programmes and awareness-raising initiatives for COVID-19 protection and mental health promotion for apartment residents in the urban Colombo Municipal Council area.^14^ Such targeted efforts are often credited for improving community understanding of, and trust in, government messaging.

#### Engagement of community and other stakeholders

Volunteer roles and organisations, including CSOs, played a key role by disseminating messages directly to households and families. In DPR Korea, the health care workforce promoted community-level health education messages.^9^ In other countries, faith leaders shared messages, played a role in dispelling stigma and observed COVID-19 guidelines in religious activities and events (eg, in Bhutan, Nepal and Sri Lanka^8 13 14^). Celebrities were engaged in Myanmar and Nepal to disseminate messages and counter misconceptions.^12 13^ New community engagement efforts included the initiation of question-and-answer sessions in Nepal, facilitated by community groups during COVID-19 district-level media briefings, which identified community concerns and priorities.^13^ Similarly, public phone hotlines, established in Myanmar and Sri Lanka to support the COVID-19 response, supported community feedback and engagement.^12 14^ Education institutions in Indonesia produced materials to support community engagement, such as a ‘community book’ to help identify and organise community activities at the district or village level.^10^

COVID-19 responses also leveraged existing formal community engagement structures, programmes and activities to improve health outcomes and health care access—ranging from community-led initiatives such as patient communities, to participatory governance platforms. In Bhutan, extensive government-led efforts to leverage community participation in health care decision-making processes at grassroots levels are framed by the concept of ‘community vitality’ (as measured against indicators of safety, donations, community relationships and family).^8^ Bhutan’s 5-year plan to decentralise governance processes to provide local governments with greater financial, planning and administrative responsibility framed community volunteering efforts and community feedback mechanisms during the COVID-19 response.^8^ Timor-Leste’s community networks also supported pandemic response efforts, where an Integrated Community Health Services (SiSCa) initiative supported engagement at a village level with a network of community health volunteers.^16^ In addition, Timor-Leste has a growing number of youth volunteer organisations focused on health promotion; these volunteer and community organisations scaled up to support COVID-19 prevention and mitigation. Quarterly ‘micro planning meetings’, conducted between health care providers and community leaders to coordinate village health plans against targets, were also useful in the pandemic response.^16^

Such community mobilisation was fundamental to the effectiveness of COVID-19 response efforts. Active and engaged communities, and community trust in government services and the health system, contributed to positive responses to government-issued messaging and effective mobilisation of community resources (eg, volunteers) to implement response measures, including targeted responses in vulnerable communities. Health literacy and trust in government influenced communities’ willingness to follow directives and mobilise as volunteers, which in turn influenced the effectiveness of different risk communication messages and community mobilisation strategies. In Thailand, VHVs played a critical role in delivering messages in local languages country-wide.^15^ Government health messages were passed to VHVs, who then provided a critical interface between health services and the community. In Sri Lanka, too, networks of peer-led groups such as Women Community Leaders in Colombo empowered community members to promote COVID-19 protective measures and health-seeking behaviours.^14^ Cultural factors enabled community solidarity—for example, the concept of *gotong royong* in Indonesia—encouraging a spirit of participation, togetherness, solidarity and synergy between individuals and communities—played a role in strengthening the community response.^10^

## Discussion

Although many countries in the South-East Asia Region have made significant progress towards improving UHC, most, like many countries globally, are unlikely to meet global targets by 2030.^6^ Reflecting the complex political, economic and social factors involved in transitioning health systems to meet the growing NCD burden and other challenges, countries are experiencing a slow pace of change. Lessons from the COVID-19 pandemic response can support orienting health systems towards PHC, and in turn accelerate progress towards UHC goals and improve health system resilience. This synthesis of 10 case studies examining PHC in the pandemic context shows how countries leveraged PHC approaches during the COVID-19 pandemic in varied ways. Key transformative approaches included use of platforms for whole-of-government action, CHW and volunteer roles integrated into primary care teams, enhanced technology-enabled models of health care and health information systems, and mechanisms that enabled community involvement in health care decision-making. These findings are consistent with other studies that identify integration of services at the primary care level, digitisation of services and advancement of community engagement as ‘game changers’ that can strengthen PHC as the foundation for resilient and equitable health systems in low-income and middle-income countries (LMICs).^21^ The synthesis draws further attention to how the crisis response leveraged innovative multisectoral and community engagement platforms that enabled collaboration across public, private, and not-for-profit organisations, CSOs, and across health care and other social sectors. Countries’ experiences add to the knowledge base by offering insights into health workforce, community engagement, digital technology, governance and financing strategies that can inform future PHC strengthening investments in the Region.

### Health workforce

Task-shifting, re-deployment of existing workforce and mobilising the community-based workforce emerged as essential strategies that addressed workforce shortages and strengthened health system capabilities during the pandemic response. Task-shifting and sharing, as strategies for optimising service delivery in resource-constrained settings through redistributing services and workforce, provide a practical model not only for emergency responses but also for the management of NCDs in LMICs.^22 23^ Integrating community-oriented workforce cadres into multidisciplinary teams is also pivotal: synthesis findings showcase how CHW and volunteer roles provided a foundation of trust and connection between government services and communities while also maintaining regular health services as part of primary care teams in the context of widespread workforce shortages. Experiences in India similarly highlight how Accredited Social Health Activists workers play a key role connecting communities with primary care services, raising awareness and engendering trust.^6^ These findings support calls for further work to strengthen enabling environments for CHWs by better integrating these roles with formal health system structures including improved systems for recognition and training.^24^ Future research should also strengthen the knowledge base on context-responsive CHW programme design and management.^25^ Findings also suggest a need to map the public health workforce and training pathways (including volunteer roles) to establish optimal workforce compositions and skill mixes to deliver integrated primary care and essential public health functions outside of hospital-centred models of care.

### Community engagement

Findings show how volunteer roles and organisations, including CSOs, facilitated and enabled government communication and coordination through well-established and emergent community engagement structures, programmes and activities. While the case studies highlight how such community mobilisation was fundamental for COVID-19 responses, these structures are also pivotal for the management of chronic conditions, enabling comprehensive care.^26^ Evidence from the Region suggests that further work is needed to identify pockets of innovation focused on community-centric approaches, such as those identified through this synthesis, to scale these for improved NCD prevention and management.^27^ Moreover, consistent with findings from other studies,^28^ the synthesis highlights how social capital underpinned community trust and engagement with government authorities during the pandemic, scaffolding community mobilisation, as well as CHW roles.

### Digital technology

Synthesis findings also draw attention to the ongoing digital transformation in the Region,^29^ while demonstrating how the pandemic accelerated the adoption and adaptation of technology-enabled models of health care and health information systems. New technology-enabled models of care, including tele-consultations, use of mobile clinics for essential medications and facilitated patient transport, may improve future NCD management, particularly in geographically dispersed, remote contexts. Synthesis findings therefore underscore a critical opportunity to identify and scale up aspects of successful digital models used in the pandemic to meet future health challenges. Others have pointed to the immense opportunities to continue to invest in digital health ecosystems, including robust, continually updated health information systems, electronic health records and telemedicine to strengthen UHC in the Region.^3 30 31^ The potential of digitalisation to support health integration, address workforce and health care access challenges and enhance surveillance in primary care must be enabled by legal and ethical frameworks to ensure patient safety, data security, and privacy and intellectual property rights as technologies are rapidly developed.^3^

### Governance for multisectoral action

The regional experience of COVID-19 also drew increased attention, as it did globally, to how societal and economic factors interact with health,^32^ with the crisis exacerbating existing social and economic disadvantage through impacts on both health and economic activity. Despite new multiagency and multilevel collaborations prompted by COVID-19, there remains an urgent need to sustain and strengthen multisectoral policy and action and to move beyond existing vertical, programmatic approaches focused on disease control and maternal and child health.^26^ Such efforts should address the optimal role of the health sector as a partner in (and potential steward of) multisectoral partnerships, and strengthen governance enablers such as policies, legislation and resourcing that incentivise collaboration, linkage infrastructure (including physical structures, as well as virtual network platforms and frameworks), shared planning including at the subnational level, and strengthened relationships and trust.^33^ Synthesis findings suggest that supportive and enabling environments for PHC allow for both centrally coordinated actions of executive government along with mechanisms to support decentralised decision-making.

### Financing integrated systems

Findings point to a need to improve public health preparedness and health system resilience by better integrating essential public health functions (including health protection, prevention and promotion) with clinical service delivery functions, which should occur well before public health emergencies arise.^33^ Despite some pockets of successful integration, most of the Region’s health systems are still oriented towards selected conditions and episodic care.^26^ Hospital-centric patterns of resource allocation, low levels of public financing and high out-of-pocket expenditures point to the need for a ‘reset’ of health financing in the Region, towards increased public investment in PHC (especially at the primary level of care).^34^

### Learning health systems

Finally, synthesis findings point to the necessity of instituting processes of continuous learning to create dynamic, adaptive health systems that respond to future health needs and crises affecting the lives of two billion people across this vast and varied Region.^1^ Without mechanisms to learn from crises such as the COVID-19 pandemic, innovations that have the potential to strengthen health systems and accelerate progress towards UHC goals risk being overlooked. Strengthening the evidence base on how to enact PHC reforms at the country level is essential.^35^ Recognising the value of evidence generation and translation, the WHO South-East Asia Regional Strategy for PHC 2022–2030 identifies the need to institutionalise learning systems for sustainable PHC as a strategic action.^1^ Learning health systems involve establishing pathways for integrating evidence into decision-making, requiring collaboration between policymakers, researchers and civil society actors in knowledge management to share relevant findings, clarify knowledge gaps and integrate evidence into policy reform processes at subnational, national and regional (across country) levels.^5 36^ The SEAR PHC Forum, established after COVID-19, represents a ‘knowledge management platform’ for capturing operational learning and driving action.^6^ These regional strategies, PHC forums and policy dialogues can be used to advocate for reforms to support community-centred PHC approaches that reflect integrated primary care with essential public health functions, multisectoral action and community empowerment.

### Strengths and limitations

The case studies offer a model of in-country-led, practice-oriented health systems research that engages policymakers as key stakeholders to reflect on pathways to improve PHC at the country level. The case studies also complement other policy-responsive reports conducted in the Region, such as the Universal Health and Preparedness Review conducted in Thailand in 2022.^37^ Strengths also include flexibility within each case study to adapt methods to account for different, country-specific local circumstances and priorities.

Limitations include an emphasis within the case studies on describing COVID-19 responses, which oriented case study recommendations towards ways to sustain or further strengthen emergency response policies and processes instituted during the pandemic. This presented challenges to the synthesis which aimed to foreground PHC-related insights and lessons. Moreover, the case studies offer very little analysis of community engagement initiatives or multisectoral policy and action beyond descriptions of selected platforms and activities. This may reflect challenges in understanding and conceptualising community empowerment and action on the social determinants of health as part of PHC,^38^ potentially suggesting a need to work towards greater conceptual clarity of PHC in practical, implementable policy terms.^39^ The use of PHC components and levers in data extraction helped to focus the synthesis on PHC, improving generalisability to other regions. Future case study research might focus more on specific enablers of PHC identified in the case studies, such as governance of multisectoral partnerships and implementing multidisciplinary team-based primary care, and to explore resourcing of policy responses to crises. Finally, while the individual case studies were authored by in-country research teams, the synthesis had no authors from case study countries outside of those from the WHO Regional Office for South-East Asia. The synthesis is strengthened by the researchers’ diverse backgrounds and use of the Astana PHC framework to minimise researcher bias in data extraction and analysis. The authors’ reflexivity statement is provided in online supplemental file 2.

## Conclusion

This article synthesises experiences and lessons from 10 country case studies conducted in the WHO South-East Asia Region examining PHC in the context of the COVID-19 pandemic. Synthesis findings demonstrate that PHC principles highlighting integrated and multidisciplinary team-based care, multisectoral policy and action, and community empowerment remain relevant for responding to public health emergencies, affirming PHC as a cornerstone of future pandemic preparedness and health system resilience.^40 41^ Strengthening PHC will require addressing health workforce shortages and maldistribution, a largely unregulated private sector, low levels of government health expenditure, hospital-based health care models and insufficient investment in prevention or attention to the social determinants of health. Findings highlight the need for robust implementation research to further examine health workforce, community engagement, digital technology and governance strategies that aim to strengthen preparedness for public health emergencies, as well as resilience in the face of evolving epidemiological and climate-related health challenges.

## Supplementary material

10.1136/bmjgh-2024-018076 online supplemental file 1

10.1136/bmjgh-2024-018076 online supplemental file 2

## Data Availability

Data sharing not applicable as no datasets generated and/or analysed for this study.
