# Supplementary material for: Primary health care in the context of the COVID-19 pandemic in 10 South-East Asian countries: a cross-case synthesis with lessons for future health systems strengthening
Source: BMJ Glob Health. 2025 Jun 30;10(Suppl 2):e018076. doi: 10.1136/bmjgh-2024-018076 (PMC12211839; doi:10.1136/bmjgh-2024-018076)
Supplement: online supplemental file 2 [file bmjgh-10-Suppl_2-s002.docx]

**Appendix S2: Reflectivity statement**

1. **How does this study address local research and policy priorities?**

This paper presents a synthesis of cross-case findings from the region’s case studies to address the question: what key lessons for PHC strengthening can be drawn from the experience of COVID-19 to inform the development of resilient health systems and accelerated progress towards UHC goals? The synthesis conducts comparative analysis across 10 case studies from the South-East Asia Region which identified lessons for PHC reform from the COVID-19 experience. The commissioning of the case studies responded to strategic priorities identified by WHO country and regional offices in 2020 and 2021.

1. **How were local researchers involved in study design?**

Each of the 10 country case studies was led and conducted by in-country author teams, who were supported by researchers in the WHO Alliance for Health Policy and Systems Research. Three PHC components framed by the 2018 Astana PHC vision were used to guide the conduct of the case studies and reporting of findings to a consistent template: 1) integrated primary care and essential public health functions; 2) multisectoral policy and action; and 3) community empowerment. The analytic framework in the synthesis used the same components. While in-country authors were not involved in the synthesis, PHC experts from the WHO Regional Office for South-East Asia were part of the synthesis authorship team.

1. **How has funding been used to support the local research team?**

The individual country case studies were commissioned and overseen by the Alliance for Health Policy and Systems Research, a hosted partnership based at WHO headquarters, in collaboration with the WHO Regional Office for South-East Asia. Case study authors used this funding to support their time to conduct the case studies.

1. **How are research staff who conducted data collection acknowledged?**

Case study reports (published separately on the WHO Alliance for Health Policy and Systems Research website) identify authorship by the research staff who conducted the case studies. In the synthesis, all country case study authors are acknowledged in the acknowledgements section of the manuscript.

1. **Do all members of the research partnership have access to study data?**

Individual case studies are published on the WHO Alliance for Health Policy and Systems Research website. All co-authors of the synthesis have access to the data extraction matrix.

1. **How was data used to develop analytical skills within the partnership?**

Case study authors were supported by experienced researchers in WHO to conduct the research, including through review of drafts and small group meetings to respond to questions. The synthesis involved input from all co-authors on all aspects of the synthesis from conceptualization to cross-case analysis and reporting.

1. **How have research partners collaborated in interpreting study data?**

The synthesis adopted a systematic data extraction process using the Astana PHC components, which enabled findings from the individual in-country-led case studies to be systematically extracted, analyzed and reported in the cross-case report.

1. **How were research partners supported to develop writing skills?**

All co-authors of the synthesis are experienced researchers. Individual case study authors were supported by experienced researchers in WHO to conduct the research, including through experienced researchers’ review of drafts and small group meetings to respond to questions.

1. **How will research products be shared to address local needs?**

Individual case studies are published on the WHO Alliance for Health Policy and Systems Research website. The published synthesis will be shared with the WHO regional and country offices with findings to be shared at relevant knowledge-sharing fora.

1. **How is the leadership, contribution and ownership of this work by LMIC researchers recognised within the authorship?**

The four authors from the WHO Regional Office for South-East Asia (ID, AS, TT, and MJ) are all LMIC-based researchers with extensive experience in PHC reform across the region. Individual case study authors are based in the countries in which their case studies were conducted.

1. **How have early career researchers across the partnership been included within the authorship team?**

AE is an early career researcher who worked closely with the in-country author teams to support the individual case studies that were used to produce the cross-case synthesis and led the synthesis. She is based in a high-income country.

1. **How has gender balance been addressed within the authorship?**

AE is the first author of the synthesis and is the only female within the authorship team. Individual case study authorship aimed to achieve an equal gender balance.

1. **How has the project contributed to training of LMIC researchers?**

The individual case studies offer a model of in-country led, practice-oriented health systems research that engage policymakers as key stakeholders to reflect on pathways to improve PHC at the country level. Case study authors were supported by experienced researchers throughout the process of conducting the case studies and subsequently with invitations to present their work at relevant knowledge-sharing fora.

1. **How has the project contributed to improvements in local infrastructure?**

This project has not directly contributed to improvements in local infrastructure.

1. **What safeguarding procedures were used to protect local study participants and researchers?**

There was no primary data collection with local participants as part of the synthesis, therefore this question is not directly applicable.
